# Automated assessment of breast arterial calcification: a new frontier in cardiovascular risk prediction

**DOI:** 10.3389/fcvm.2026.1860391

**Published:** 2026-09-18

**Authors:** Lingling Wei, Zhebing Wu, Liangyun Wu, Li Jiang

**Affiliations:** 1 Department of Cardiology, Dongyang Traditional Chinese Medicine Hospital, Jinhua, Zhejiang Province, China; 2 Department of Stomatology, Dongyang Traditional Chinese Medicine Hospital Jinhua City, Jinhua, Zhejiang Province, China; 3 Department of Breast Surgery, Dongyang Maternal and Child Health Hospital, Jinhua, Zhejiang Province, China

**Keywords:** artificial intelligence, breast arterial calcification, cardiovascular risk stratification, coronary artery disease, hypertension

## Abstract

Breast Arterial Calcification (BAC) is a common incidental finding on mammograms, traditionally regarded as a benign degenerative change. However, a growing body of evidence indicates that BAC is associated with coronary artery disease (CAD), particularly in women with hypertension. Hypertension may contribute to BAC through persistent hemodynamic stress and vascular endothelial dysfunction, which promote the osteoblast-like transformation of medial vascular smooth muscle cells and calcium deposition in the extracellular matrix. The resulting increase in arterial stiffness is consistent with Mönckeberg's medial calcification, a process distinct from atherosclerotic plaque calcification. Epidemiological data indicate that individuals with BAC have a higher risk of CAD than those without BAC. This association may be stronger in individuals with elevated blood pressure and may provide information beyond traditional Framingham risk scores. In clinical practice, routine mammography can readily detect BAC. Emerging deep learning-based artificial intelligence (AI) algorithms may improve the consistency and sensitivity of BAC assessment, providing a non-invasive, cost-effective, and widely accessible approach to cardiovascular screening in hypertensive women. Current multicenter cohort studies are beginning to integrate BAC scores into cardiovascular risk prediction models and to examine their relationships with blood pressure control and anti-calcification therapies. Future studies should validate AI algorithms across equipment and populations, clarify the relationships of BAC with blood pressure variability, micro-inflammation, and myocardial perfusion, and evaluate interventions targeting medial calcification.

## 1 Introduction

In the realm of cardiovascular health, the early identification and stratification of individuals at risk for coronary artery disease (CAD) remain paramount for effective prevention and timely intervention (1). Traditional risk assessment models, while valuable, often exhibit limitations, particularly in women, where the manifestation and progression of cardiovascular disease can differ significantly from men (2). A serendipitous finding on routine breast imaging, breast arterial calcification (BAC), has emerged as a compelling, yet often overlooked, indicator of systemic vascular health (3). Historically, BAC was largely dismissed as an innocuous age-related degenerative change, a mere incidental observation on mammograms performed primarily for breast cancer screening (4–6). Accumulating evidence links the presence and extent of BAC to CAD, major adverse cardiovascular events (MACE), and cardiovascular mortality (7–12). Mammography may therefore complement breast cancer screening by providing additional information on cardiovascular risk in women.

The association between BAC and CAD may partly reflect shared pathophysiological mechanisms, with hypertension as a potential mediator. Chronic hemodynamic stress contributes to vascular remodeling and calcification (13–16). Atherosclerotic calcification primarily affects the intima, whereas BAC predominantly represents medial calcification, often referred to as Mönckeberg's arteriosclerosis. Although anatomically distinct, these processes may share pathways influenced by systemic factors such as hypertension (17, 18). Clarifying how hypertension contributes to BAC formation and progression, and how BAC reflects systemic arterial disease, may improve cardiovascular risk assessment in women.

Artificial intelligence (AI) and machine-learning technologies may improve the detection, quantification, and clinical application of BAC. Manual assessment of BAC on mammograms is subjective, time-consuming, and affected by inter-observer variability, which limits its routine clinical use (19). AI-powered algorithms can automate and standardize BAC detection and quantification, providing objective measurements that may be integrated into existing imaging workflows (20–23). These tools may help transform BAC from an incidental finding into a reproducible cardiovascular imaging biomarker, particularly in high-risk groups such as hypertensive women. As a narrative review, this article summarizes current evidence on the relationship between BAC and hypertension, with emphasis on the hemodynamic, inflammatory, and molecular mechanisms underlying BAC formation and progression. It also reviews the associations of BAC with CAD and other atherosclerotic cardiovascular diseases and discusses emerging AI-assisted applications in BAC detection, quantitative assessment, and cardiovascular risk prediction.

## 2 Breast arterial calcification (BAC) fundamentals & epidemiology

BAC represents a distinct form of vascular calcification characterized by the deposition of calcium salts within the medial layer of the breast arteries, primarily the small and medium-sized arteries (5, 6). This process is histologically distinct from atherosclerotic calcification, which typically involves the intimal layer of larger arteries and is associated with lipid accumulation, inflammation, and plaque formation. While atherosclerotic calcification is a hallmark of CAD and other forms of atherosclerotic cardiovascular disease (ASCVD), BAC is considered a manifestation of Mönckeberg's arteriosclerosis, a non-occlusive calcification of the arterial media. Despite these histological differences, a growing body of evidence suggests a significant clinical association between BAC and systemic atherosclerosis, including CAD, indicating shared underlying risk factors and potentially common pathways of vascular injury and repair (10, 11).

The detection of BAC is typically an incidental finding on routine mammography, a widely utilized screening tool for breast cancer in women (6). Mammograms, being two-dimensional x-ray images, can clearly visualize the linear or curvilinear calcifications within the arterial walls of the breast parenchyma. The prevalence of BAC varies significantly across different populations and age groups, generally increasing with advancing age. Early studies, such as that by Kataoka et al. (24), reported a prevalence of 16.0% in women older than 55 years, highlighting its commonality in older populations. More recent studies and meta-analyses have shown a broader range, with prevalence rates reported from 3% to 29% in various cohorts, and an average prevalence of around 10% in a large meta-analysis of over 33,000 women (10, 11). A retrospective study of 228 women by Reddy et al. showed a prevalence of 39% for BAC, and that women with BAC were older, more likely to be Hispanic, and postmenopausal (25). This age-dependent increase is a consistent finding across numerous studies, suggesting a cumulative effect of aging and chronic exposure to risk factors on vascular calcification (15, 26, 27).

Beyond age, several traditional cardiovascular risk factors have been consistently associated with the presence of BAC. Hypertension and diabetes mellitus are among the most robustly linked conditions (13, 14). Çetin et al. demonstrated a significantly higher prevalence of BAC in diabetic patients and hypertensive patients compared to non-diabetic, non-hypertensive controls, with the prevalence increasing with age in all groups. Similarly, Yildiz et al. identified age and metabolic syndrome as independent predictors of BAC, with high blood pressure and low high-density lipoprotein (HDL) levels being particularly significant components of metabolic syndrome associated with BAC (15). Fathala et al. (2018) also found a strong association between BAC and age, diabetes, hypertension, and chronic kidney disease in asymptomatic Saudi women (27). These findings are further corroborated by Al-Mohaissen et al. (2022), who reported positive associations between breast calcifications (including BAC) and hypertension, diabetes, and hypercholesterolemia in women aged 40 years and older, even after controlling for age and body mass index (BMI) (16).

Interestingly, while hyperlipidemia is a major risk factor for atherosclerotic disease, its association with BAC has been less consistent across studies. Lee et al. (2020) in a systematic review and meta-analysis found that BAC was associated with hypertension and diabetes mellitus but not with hypercholesterolemia (11). This discrepancy might reflect the distinct pathological nature of medial calcification compared to intimal atherosclerosis, where lipid accumulation plays a more central role. Conversely, smoking has shown an inverse association with BAC in some studies (11, 24), a finding that warrants further investigation as it contrasts with its well-established pro-atherogenic effects. This inverse relationship could potentially be explained by the higher mortality rates among smokers with severe cardiovascular disease, leading to a survivor bias in cross-sectional studies, or by complex interactions with other risk factors.

Reproductive factors, including parity and breastfeeding, have also been examined, although findings remain inconsistent. Yalçın Şafak et al. found that age, parity, and breastfeeding history were independently associated with BAC in a Turkish cohort, suggesting a possible hormonal influence on vascular calcification (26). Bielak et al. reported associations between BAC, parity, and age at first birth, raising the possibility that lifetime hormonal exposure may influence BAC in postmenopausal women (28). However, the underlying mechanisms remain uncertain. Overall, hypertension and diabetes are among the most consistently reported cardiovascular correlates of BAC, with hypertension repeatedly identified as an independent predictor. This association provides the rationale for examining the role of hypertension in BAC formation and progression.

## 3 Hypertension and BAC: pathophysiological links

### 3.1 Mechanical stress and vascular smooth muscle cell transformation

The association between hypertension and BAC may be mediated by chronic mechanical stress and the cellular responses it induces in the arterial wall. BAC predominantly represents medial calcification, whereas atherosclerotic calcification is mainly intimal. Nevertheless, both processes involve regulated vascular injury and repair pathways influenced by systemic factors such as hypertension. Hypertension promotes medial stiffening and calcium deposition. This is an active osteogenic process involving vascular smooth muscle cells (VSMCs) phenotypic transformation and dysregulation of calcification inhibitors (6) (Figure 1).

**Figure 1:**
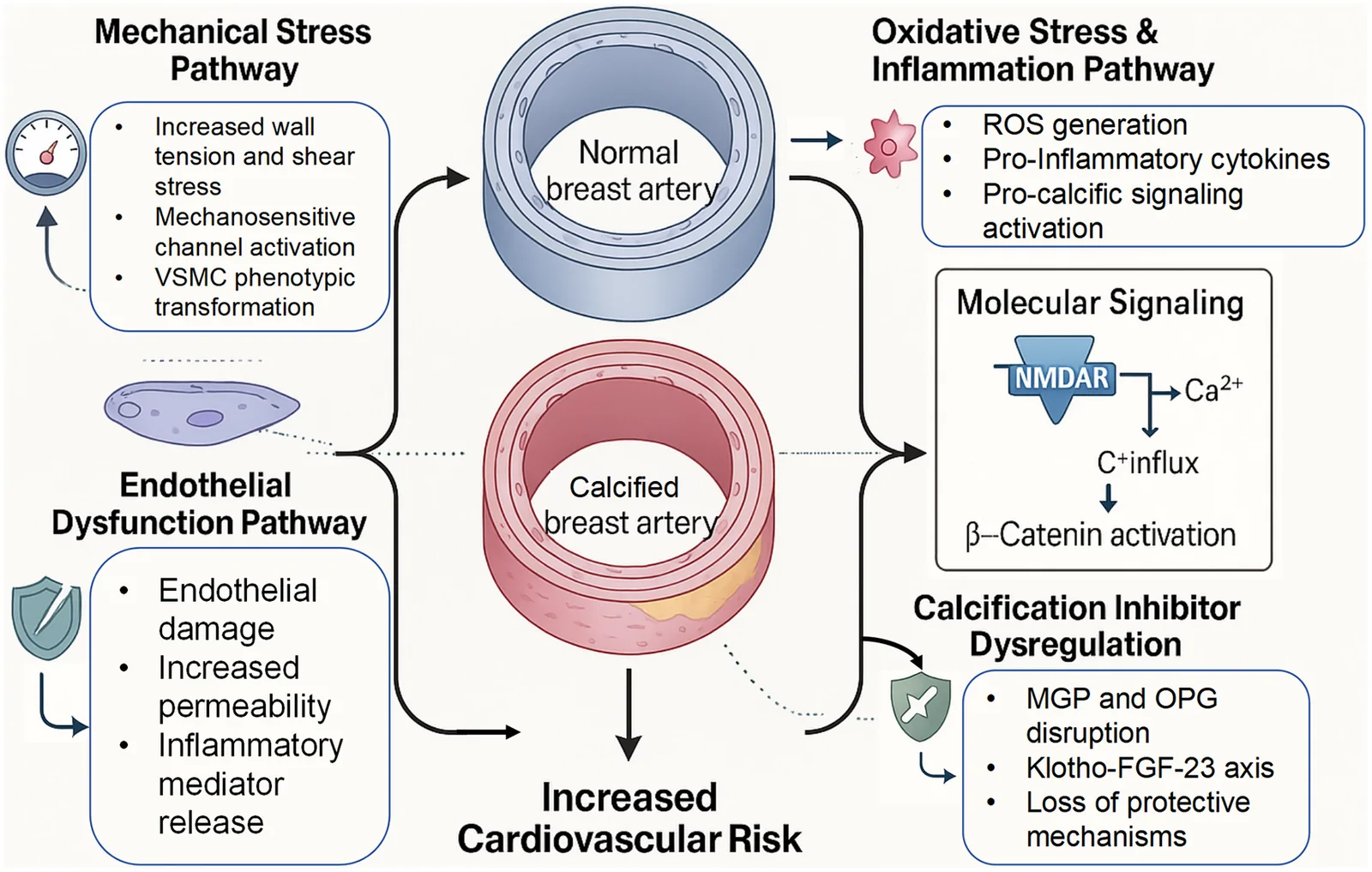
Pathophysiological mechanisms linking hypertension to breast arterial calcification. Schematic representation of the four major pathways contributing to breast arterial calcification: mechanical stress, oxidative stress and inflammation, endothelial dysfunction, and calcification inhibitor dysregulation. The mechanical stress pathway involves increased wall tension and shear stress, leading to mechanosensitive channel activation and vascular smooth muscle cell (VSMC) phenotypic transformation. The oxidative stress pathway is characterized by reactive oxygen species (ROS) generation, pro-inflammatory cytokine production, and pro-calcific signaling activation. Endothelial dysfunction results in endothelial damage, increased permeability, and inflammatory mediator release. Calcification inhibitor dysregulation involves matrix Gla protein (MGP) and osteoprotegerin (OPG) disruption, Klotho-FGF-23 axis abnormalities, and loss of protective mechanisms. These pathways converge on molecular signaling involving NMDAR-mediated calcium influx and β-catenin activation, ultimately leading to medial calcification in breast arteries (shown in yellow deposits) and increased cardiovascular risk.

Persistent hemodynamic stress is a major driver of vascular structural and functional alterations in hypertension. Chronic hypertension increases shear stress, circumferential stress, and tensile stress within the arterial wall. These forces act directly on VSMCs and the extracellular matrix (ECM) within the arterial media and may trigger VSMC phenotypic transformation. Under physiological conditions, VSMCs maintain a contractile phenotype that supports vascular tone and structural integrity. In hypertension, however, VSMCs may dedifferentiate toward a synthetic or osteoblast-like phenotype and express bone-associated proteins, including alkaline phosphatase (ALP), osteocalcin, and osteopontin (29, 30). This transition promotes calcium-binding protein production and calcium deposition within the ECM (6).

### 3.2 Cellular and molecular mechanisms of hypertension-induced calcification

Several cellular and molecular mechanisms underpin hypertension-induced calcification (29, 30). One key concept is the “kite effect,” where increased wall tension and stiffness, driven by hypertension, create an environment conducive to calcium deposition (31). Mechanical strain on the arterial wall can directly activate mechanosensitive channels and signaling pathways within VSMCs, leading to gene expression changes that favor calcification (32). High pressure can induce oxidative stress and inflammation within the vascular wall, both potent drivers of VSMC phenotypic modulation and calcification (14). Reactive oxygen species (ROS) generated under hypertensive conditions can activate pro-calcific signaling pathways, while inflammatory cytokines can directly stimulate osteogenic differentiation of VSMCs (13, 33).

Furthermore, hypertension often coexists with other metabolic derangements, such as diabetes and chronic kidney disease (CKD), which independently contribute to vascular calcification and synergistically amplify high blood pressure effects (15, 16, 26, 32, 34). In CKD, dysregulation of mineral metabolism, including hyperphosphatemia and elevated fibroblast growth factor 23 (FGF-23) levels, plays a significant role in promoting vascular calcification (32). Wungu et al. (2024) conducted a meta-analysis showing that FGF-23 was positively correlated with arterial calcification, thickness, and stiffness, while Klotho, a co-receptor for FGF-23, was inversely correlated with arterial calcification and carotid intima-media thickness (CIMT) (32). This suggests the Klotho-FGF-23 axis is a critical regulator of arterial remodeling and calcification, and its dysregulation, often seen in CKD and potentially exacerbated by hypertension, contributes to calcific burden.

### 3.3 Endothelial dysfunction and calcification inhibitor balance

The interaction of elevated pressure, endothelial dysfunction, and inflammation also promotes medial calcification (2, 35). Hypertension damages the endothelial lining, leading to increased permeability and pro-inflammatory mediator release (36). This local inflammation further promotes VSMC dedifferentiation and osteogenic transformation (15). Moreover, loss of endogenous calcification inhibitors, such as matrix Gla protein (MGP) and osteoprotegerin (OPG), represents a critical factor (1). Warfarin, an anticoagulant that inhibits vitamin K-dependent proteins like MGP, has been shown to increase peripheral arterial calcification, particularly medial calcification, in both men and women, underscoring the importance of these inhibitors in preventing vascular calcification (33, 37).

Hypertension may promote BAC through direct mechanical stress, associated metabolic disorders such as chronic kidney disease (CKD), and pro-calcific molecular pathways, including NMDAR/Ca2+/β-catenin signaling related to glutamate metabolism (13, 14, 29, 34). Together, these mechanisms favor calcium deposition in the arterial media and may link mammographic BAC with arterial stiffness and systemic vascular pathology (8, 9).

## 4 BAC as marker of CAD

The growing body of evidence strongly suggests that BAC, despite its distinct medial localization, serves as a significant marker for the presence and severity of CAD and other ASCVD (10, 11). This association has been explored through various study designs, including cross-sectional (38), cohort (39), and meta-analytic approaches (10, 27), yielding largely consistent findings that underscore BAC's potential utility in cardiovascular risk stratification, particularly in women (4, 19) (Table 1).

**Table 1:** Selected evidence on the associations of breast arterial calcification with coronary artery disease and cardiovascular outcomes.

Study	Study design and population	Outcome assessed	Quantitative findings	Interpretation	References
Kataoka et al.	Cross-sectional screening study of women without clinically apparent cardiovascular disease	Prevalent coronary heart disease	Age-adjusted OR, 2.54	BAC was associated with prevalent coronary heart disease, although its sensitivity as an isolated screening marker was limited.	(24)
Lee et al.	Systematic review and meta-analysis; 31 studies involving 35,583 women	Presence of CAD	Overall OR, 2.61 (95% CI, 2.12–3.21); moderate-to-severe BAC: OR, 4.83 (95% CI, 1.50–15.54) using a 4-point scale and OR, 2.95 (95% CI, 1.49–5.84) using a 12-point scale	BAC was associated with CAD, with stronger associations observed for more severe BAC; substantial heterogeneity was present.	(11)
Osman et al.	Systematic review and meta-analysis; 18 studies involving 33,494 women	Presence of CAD	Unadjusted OR, 2.14 (95% CI, 1.63–2.81); adjusted OR, 2.39 (95% CI, 1.68–3.41); both *p* < 0.001	BAC remained associated with CAD after adjustment for conventional cardiovascular risk factors.	(10)
Iribarren et al.	Prospective cohort study; 5,059 postmenopausal women	Incident hard ASCVD and global CVD	Hard ASCVD: adjusted HR, 1.51 (95% CI, 1.08–2.11; *p* = 0.020); global CVD: adjusted HR, 1.23 (95% CI, 1.002–1.52; *p* = 0.048)	BAC was independently associated with incident cardiovascular outcomes and provided incremental risk-stratification information.	(7)
Rose et al.	Retrospective AI-based analysis; 99 women with mammography and chest CT	MACE and moderate-to-high ASCVD risk	Per 1-unit increase in AI-derived BAC score: OR, 1.02 for MACE (*p* = 0.005) and OR, 1.06 for moderate-to-high ASCVD risk (*p* = 0.010); MACE risk was 17% higher in BAC-positive women (95% CI, 8%–33%)	Higher AI-derived BAC scores were associated with greater cardiovascular risk, although the study sample was small.	(12)
Huang et al.	Single-center observational study; 213 women undergoing mammography and deep learning-based CCTA assessment	CAD-RADS ≥3	Adjusted OR for BAC presence, 10.22 (95% CI, 2.86–36.49; *p* < 0.001); PPV, 68.2% (95% CI, 45.1%–86.1%); NPV, 91.6% (95% CI, 86.8%–95.1%)	BAC was independently associated with higher CAD-RADS categories, but the estimate had a wide confidence interval.	(43)
Fathala et al.	Retrospective study; 435 women undergoing mammography and myocardial perfusion SPECT	Myocardial ischemia	Total BAC score: *p* = 0.210; BAC severity category: *p* = 0.307	Neither BAC burden nor BAC severity was associated with myocardial ischemia on myocardial perfusion imaging.	(40)
Fathala et al.	Retrospective single-center study; 203 women undergoing mammography and invasive coronary angiography	Obstructive CAD on invasive coronary angiography	Association between BAC and abnormal angiography: *p* = 0.108	BAC did not predict angiographically documented obstructive CAD in this selected population.	(41)

Effect estimates are presented as reported in the original studies. OR and HR values are shown with 95% CIs when available.

BAC, breast arterial calcification; ASCVD, atherosclerotic cardiovascular disease; CAD, coronary artery disease; CCTA, coronary computed tomography angiography; CI, confidence interval; CT, computed tomography; CVD, cardiovascular disease; HR, hazard ratio; MACE, major adverse cardiovascular events; NPV, negative predictive value; OR, odds ratio; PPV, positive predictive value; SPECT, single-photon emission computed tomography.

Early investigations into the relationship between BAC and CAD provided foundational insights. Kataoka et al. observed that BAC was associated with prevalent coronary heart disease (CHD) after adjustment for age, with an odds ratio of 2.54, suggesting that BAC could provide additional information for identifying cardiovascular disease risk among otherwise healthy women (24). This early finding, despite a low sensitivity, highlighted the potential of BAC as a non-invasive indicator.

Subsequent studies and meta-analyses have significantly strengthened this association. A comprehensive systematic review and meta-analysis by Lee et al., encompassing 31 studies and over 35,000 women, demonstrated a robust association between BAC and CAD (11). This meta-analysis further revealed that the association was stronger with moderate-severe BAC compared to mild BAC, suggesting a dose-response relationship between the extent of BAC and CAD presence. Another meta-analysis by Osman et al., which included 18 studies and nearly 33,500 women, similarly found a statistically significant association between BAC and CAD, reinforcing BAC as an independent predictor of CAD (10). These meta-analyses provide compelling evidence for the consistent and independent predictive value of BAC for CAD across diverse populations.

Although the overall evidence supports an association between BAC and CAD, several individual studies have reported more nuanced findings. After adjustment for age, Abedi et al. found no significant association between the presence or severity of BAC and either coronary artery calcium (CAC) score or coronary artery stenosis (38). Fathala et al. reported that BAC did not predict myocardial ischemia on myocardial perfusion single-photon emission computed tomography (MPS) and therefore did not support additional cardiac investigation based solely on BAC (40). Pudil et al. did not confirm BAC as a general predictor of CAD, although BAC was associated with more severe coronary atherosclerosis, including triple-vessel disease (39). In a separate retrospective study of Saudi women, Fathala et al. found no association between visually detected BAC and angiographically proven obstructive CAD. However, BAC-positive women were older and more frequently had diabetes and hypertension, suggesting that BAC may reflect cumulative cardiovascular risk rather than directly predict obstructive CAD in all populations (41). These differences may reflect variation in study populations, BAC definitions and quantification methods, and cardiovascular endpoints, such as angiographic disease vs. clinical events. They may also reflect the distinction between medial BAC, which is associated with arterial stiffness and vascular aging, and intimal coronary atherosclerosis, although the two processes may coexist and share risk factors.

Beyond the presence of CAD, BAC has also been linked to MACE. Iribarren et al. conducted a cohort study of over 5,000 postmenopausal women and found that BAC presence was associated with a 1.51-fold increased hazard of hard atherosclerotic CVD events and a 1.23-fold increased hazard of global CVD events, even after adjusting for traditional CVD risk factors (7). Their study further demonstrated that BAC status provided additional risk stratification beyond the Pooled Cohort Equations (PCE) risk, leading to improvements in model calibration and reclassification of ASCVD risk. This suggests that BAC acts as a “risk-enhancing factor,” providing incremental predictive value beyond conventional risk scores. Rose et al. utilized an AI detection model for BAC and found that a one-unit increase in BAC score resulted in a 2% increased odds of having MACE and a 6% increased odds of having moderate to high ASCVD risk (>7.5%), further supporting BAC's association with adverse cardiovascular outcomes (12).

An important unresolved question is whether BAC provides incremental prognostic information beyond that already captured by CAC. Previous studies have shown that BAC is associated with greater CAC burden and may also be associated with the progression of CAC and coronary atherosclerotic plaque (42, 43). Because BAC predominantly reflects medial arterial calcification, whereas CAC primarily represents calcification within intimal atherosclerotic plaques, the two markers may capture partly distinct vascular pathological processes (34). Their combined assessment may therefore provide complementary cardiovascular risk information. However, current evidence has focused mainly on associations of BAC with CAC burden or coronary atherosclerosis progression rather than on incremental prediction of clinical events (42, 43). Whether adding BAC to a model that already includes clinical risk factors and CAC improves MACE prediction remains uncertain and should be evaluated in longitudinal studies.

Experimental and mechanistic studies suggest that medial and intimal calcification, although anatomically distinct, share several regulated cellular pathways. In both settings, VSMCs may lose their contractile phenotype and acquire osteogenic characteristics, with apoptosis, inflammatory and oxidative stress signaling, extracellular vesicle release, and ECM remodeling promoting calcium-phosphate deposition (44). Experimental induction of chronic VSMCs apoptosis has also been shown to accelerate atherosclerosis while promoting both plaque calcification and medial degeneration (45). These findings support biological overlap between medial arteriosclerosis and calcified atherosclerotic plaque formation, although their initiating factors and clinical consequences remain distinct.

The relationship between BAC and CAD risk factors is also well-established. As discussed in the epidemiology section, BAC is consistently associated with age, hypertension, diabetes mellitus, and metabolic syndrome (13–15, 26, 27). These associations reinforce the notion that BAC is not an isolated phenomenon but rather a visible manifestation of systemic vascular aging and cumulative exposure to cardiovascular risk factors (46). Huang et al. demonstrated a significant correlation between the severity of CAD, as evaluated by deep learning-based coronary computed tomography angiography (CCTA) CAD-RADS, and the presence of BAC on mammography. They found that BAC was significantly higher in the high CAD-RADS group and remained a significant independent predictor of CAD-RADS severity after adjustment for other variables, suggesting its utility in predicting the extent of coronary atherosclerosis (47).

Overall, although BAC represents medial calcification, its association with CAD and MACE, particularly in meta-analyses and studies using advanced coronary imaging, supports its potential as a women-specific cardiovascular biomarker. Its incidental detection on routine mammography and possible incremental value beyond traditional risk scores warrant further evaluation. However, inconsistent findings regarding angiographic CAD severity indicate that standardized BAC quantification and larger prospective studies are required before BAC can be incorporated into routine cardiovascular risk assessment.

## 5 Role of AI in quantifying BAC and predicting CAD

On mammography, BAC typically appears as smooth linear or curvilinear radiopaque deposits following the course of breast arteries; when both arterial walls are calcified, the characteristic parallel “tram-track” appearance may be seen (Figure 2) (6, 9). Qualitative assessment commonly records BAC as absent or present and may assign an ordinal visual severity grade. Semiquantitative approaches estimate BAC burden according to features such as the number of calcified vessels, vessel-wall opacification, and the total length of calcified arterial segments (19, 48). Quantitative approaches provide continuous measurements, including calcium mass derived from full-field digital mammography or algorithm-derived calcification burden obtained after automated vessel segmentation (20–23, 54). However, no universally accepted BAC assessment or reporting system has yet been established, which limits direct comparison across studies.

**Figure 2:**
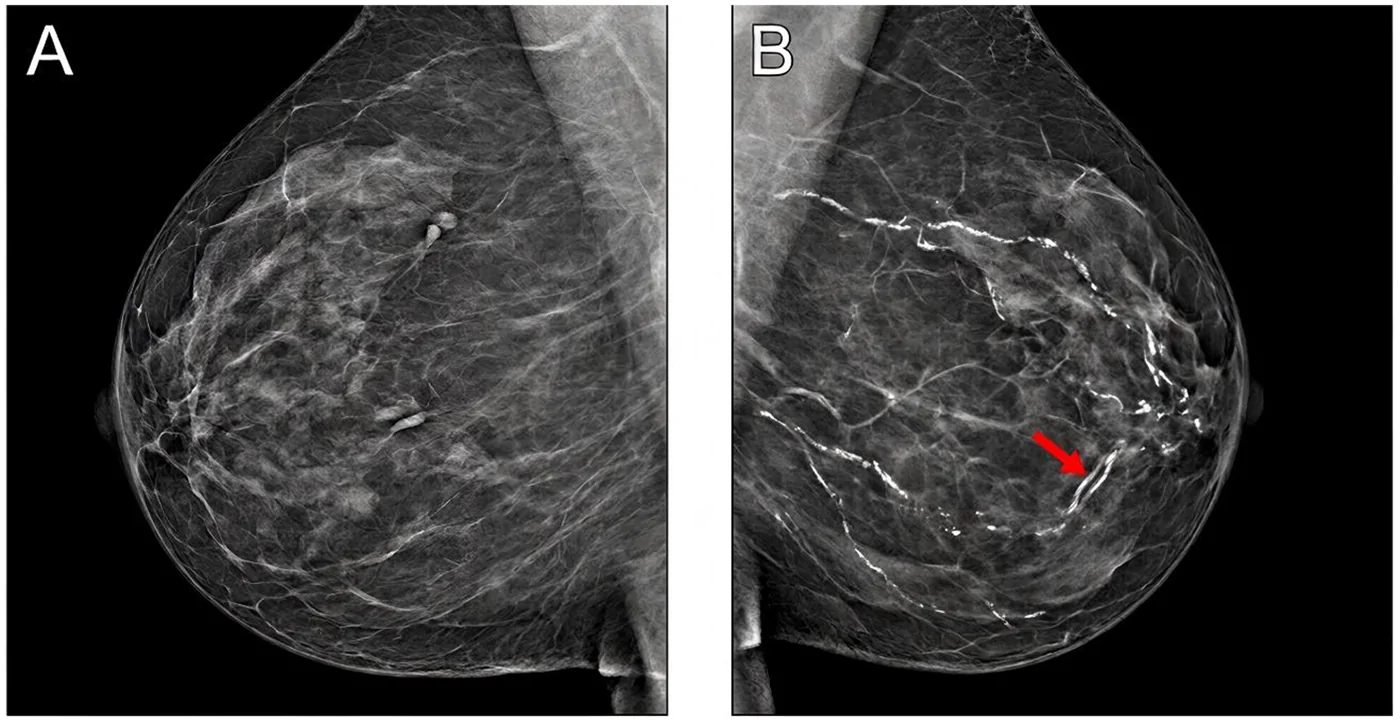
Representative mammographic appearance of breast arterial calcification. **(A)** Mediolateral oblique mammogram without radiographic evidence of breast arterial calcification. No linear or curvilinear calcifications following the expected course of the breast arteries and no parallel “tram-track” calcifications are identified. Because noncalcified breast arteries are generally not conspicuous on routine mammography, this image serves as a BAC-negative mammographic comparison rather than as direct visualization of a normal noncalcified artery. **(B)** Mediolateral oblique mammogram demonstrating typical breast arterial calcification, characterized by linear and curvilinear radiopaque calcifications following the course of multiple breast arteries. The red arrow highlights a representative segment showing parallel calcification of both arterial walls, producing the characteristic “tram-track” appearance. BAC, breast arterial calcification.

Manual BAC assessment is subjective, time-consuming, and insufficiently standardized, which limits its routine use as a cardiovascular risk marker (5, 19, 23). Differences among qualitative and semiquantitative methods also contribute to inter-observer variability and reduce comparability across studies. AI and machine learning (ML), particularly deep learning, may address these limitations through automated BAC detection, segmentation, and quantification (49) (Table 2).

**Table 2:** Artificial intelligence applications in breast arterial calcification assessment and cardiovascular risk evaluation.

Study	AI application	Dataset and target	Quantitative performance or association	Potential clinical application	Main limitation	References
AlJabri et al.	Recurrent attention U-Net for BAC segmentation	2,000 synthesized 2D mammograms; pixel-level BAC segmentation	Accuracy, 99.9%; sensitivity, 69.6%; precision, 68.4%; F1 score, 66.6%; Jaccard index, 59.5%	Automated BAC localization and quantitative segmentation	Evaluation was based on synthesized 2D mammograms, and sensitivity remained moderate.	(20)
Watanabe et al.	Commercial deep learning software for binary BAC detection	314 digital mammography examinations from 253 women and 277 digital breast tomosynthesis examinations from 143 women	Digital mammography: AUC, 0.96 (95% CI, 0.93–0.98), sensitivity 87.0%, specificity 92.0%, accuracy 91.0%; digital breast tomosynthesis: AUC, 0.95 (95% CI, 0.92–0.98), sensitivity 88.0%, specificity 90.0%, accuracy 89.0%	Assistance with routine BAC detection and standardized reporting	Retrospective test sets were relatively small and obtained using a single equipment manufacturer.	(21)
Saccenti et al.	Deep learning software for BAC detection and quantification	502 women with digital mammography and CT-based CAC assessment	Correlation between AI and manual BAC scores: r = 0.83; for marked CAC: sensitivity 32.7% (95% CI, 24.2%–42.2%), specificity 96.1% (95% CI, 93.7%–97.8%), PPV 71.2%, NPV 83.1%, and accuracy 81.9%	Objective BAC quantification and identification of women likely to have marked CAC	High specificity was accompanied by low sensitivity for marked CAC.	(22)
Rose et al.	AI-derived quantitative BAC scoring	99 women with mammography, chest CT, and cardiovascular outcome data	Per 1-unit increase in BAC score: OR, 1.02 for MACE (*p* = 0.005) and OR, 1.06 for moderate-to-high ASCVD risk (*p* = 0.010)	Opportunistic cardiovascular risk assessment using routinely acquired mammograms	Small retrospective cohort and limited adjustment for potential confounding factors	(12)
Huang et al.	Deep learning-based CAD-RADS assessment on CCTA combined with visually assessed BAC	213 women undergoing mammography and CCTA; target outcome was CAD-RADS ≥3	Adjusted OR for BAC presence, 10.22 (95% CI, 2.86–36.49; *p* < 0.001); PPV, 68.2%; NPV, 91.6%	Linking mammographic BAC with automated assessment of coronary disease severity	BAC itself was assessed visually rather than by AI, and the study was single-center with few BAC-positive cases.	(43)

AI performance metrics are reported according to the target task of each study and should not be directly compared across segmentation, binary detection, quantification, and outcome-association studies. AUC values are reported to two decimal places, and percentages are reported to one decimal place where source data permit.

AI, artificial intelligence; ASCVD, atherosclerotic cardiovascular disease; AUC, area under the receiver operating characteristic curve; BAC, breast arterial calcification; CAC, coronary artery calcification; CAD-RADS, Coronary Artery Disease Reporting and Data System; CCTA, coronary computed tomography angiography; CI, confidence interval; CT, computed tomography; MACE, major adverse cardiovascular events; NPV, negative predictive value; OR, odds ratio; PPV, positive predictive value.

### 5.1 Deep learning models for BAC detection and quantification

Deep learning models, particularly convolutional neural networks (CNNs), are widely used for medical image segmentation, detection, and classification (50–53). For BAC assessment, these models can learn mammographic patterns, identify subtle calcifications, and quantify calcification burden. AlJabri et al. developed a recurrent attention U-Net model for BAC segmentation and quantification on synthesized two-dimensional mammograms. By incorporating recurrent mechanisms and attention modules, the model achieved high overall accuracy, sensitivity, and F-1 score, supporting the feasibility of automated BAC identification (20). Further external validation is nevertheless required before such models can support standardized clinical reporting.

Several other AI-based approaches have been developed for BAC detection and quantification. Chidurala et al. provided a comprehensive review of AI models for BAC detection, highlighting models like DU-Net, difference-of-Gaussian generative adversarial network (DoG-GAN), and Simple Context U-Net (SCU-Net), which utilize U-Net architectures optimized for segmentation. These models have shown performance metrics that rival or surpass human experts, improving BAC detection reliability and scalability (23). AI's ability to precisely segment and quantify calcium mass, as explored by Molloi et al. using densitometry techniques on digital mammography, further underscores potential for objective measurement beyond mere presence/absence assessment (54). While Molloi's work predates the deep learning revolution, it established groundwork for quantitative assessment that AI can now automate and refine.

### 5.2 Clinical implementation and performance validation

The integration of AI-driven BAC assessment into clinical workflows shows significant promise for improving cardiovascular risk prediction. Watanabe et al. evaluated commercially available AI-based software for BAC detection on both digital mammography (DM) and digital breast tomosynthesis (DBT) examinations. The software demonstrated promising performance with high AUCs (0.96 for DM, 0.95 for DBT), suggesting potential to aid radiologists in consistent BAC detection and reporting (21). Similarly, Saccenti et al. evaluated deep learning-based software for automatic BAC detection and quantification, demonstrating very strong correlation between AI-generated BAC score and manual BAC score (*r* = 0.83). This automated BAC AI score showed potential utility in diagnosing marked coronary artery calcification (CAC) and could promote BAC integration into mammography reports, thereby improving awareness of women's cardiovascular risk status (22).

### 5.3 AI-enhanced cardiovascular risk assessment integration

Beyond detection, AI contributes to sophisticated cardiovascular risk assessment by integrating BAC data with other clinical parameters. Rose et al. utilized an AI detection model for BAC and found that AI-generated BAC measurements were associated with MACE and useful in corroborating ASCVD risk, suggesting these measurements could supplement widely adopted ASCVD and CAC scores to identify and risk-stratify women at increased CVD risk (12). This highlights AI's capacity not only to identify biomarkers but to integrate them into broader predictive frameworks. Huang et al. demonstrated significant correlation between deep learning-based CAD-RADS (from CCTA) and BAC on mammography, indicating AI's role in linking different imaging modalities for comprehensive risk assessment (47).

The broader field of AI in cardiovascular risk prediction is developing rapidly. Machine-learning methods, including random forests, logistic regression, gradient boosting machines, and neural networks, can model complex interactions among cardiovascular risk factors and have shown improved predictive performance compared with some established algorithms (55–58). Dalal et al. reported an accuracy of 99.1% for cardiovascular disease prediction using a Kaggle dataset (59). Dritsas and Trigka reported an accuracy of 87.8% and an area under the receiver operating characteristic curve (AUC) of 98.2% after feature selection and synthetic minority oversampling technique (SMOTE)-based processing, with a stacking ensemble providing the best performance (60). Zaidi et al. developed HeartEnsembleNet, which achieved 92.95% accuracy and 93.08% precision in a dataset of 70,000 patients with cardiac disease (61). Oikonomou and Khera further reviewed applications of machine learning in precision diabetes care and cardiovascular diagnosis, prognostication, phenotyping, and treatment (62). Although these studies are not specific to BAC, they provide contextual evidence for the broader potential of AI-based cardiovascular risk prediction. AI-derived imaging biomarkers, such as epicardial fat measurements from computed tomography (CT), also demonstrate the potential value of extracting cardiovascular information from routinely acquired images (63).

AI's potential extends to various risk prediction aspects, including wearable sensor data (64), carotid plaque scores (65), polygenic risk scores (66), stress hyperglycemia ratios (67), calcium-omics (68), and liver fibrosis markers (69). AI's ability to process and synthesize diverse data types, including imaging biomarkers like BAC alongside clinical and genetic information, promises more holistic and precise cardiovascular risk stratification approaches.

Widespread adoption of AI-based BAC assessment requires model generalizability across mammography systems and diverse patient populations, supported by large-scale multicenter external validation (23, 70–73). Safety, ethical considerations, algorithmic bias, transparency, and explainability must also be addressed. Explainable artificial intelligence (XAI) may help clinicians understand model outputs, thereby supporting trust and clinical decision-making (70). If these challenges are addressed, AI could facilitate the transition of BAC from an underreported incidental finding to a standardized and clinically useful cardiovascular imaging biomarker.

## 6 Discussion

### 6.1 Clinical application significance and guideline recommendations

The strong association between BAC and CAD, particularly in hypertensive women, supports integrating BAC assessment into routine cardiovascular risk stratification. Mammography offers a unique, cost-effective opportunity to simultaneously assess cardiovascular health without additional patient visits, radiation exposure, or financial burden (5, 6, 19) (Table 3).

**Table 3:** Current evidence and remaining barriers to the clinical implementation of breast arterial calcification assessment.

Aspect	Current evidence	Quantitative evidence	Potential clinical implication	Remaining limitation	References
Reporting practice	BAC reporting remains non-standardized and is commonly limited to presence or absence.	Not applicable; no universally accepted quantitative BAC threshold or reporting scale has been established.	Standardized terminology and severity grading could improve communication and study comparability.	Lack of consensus regarding scoring methods, thresholds, and reporting responsibility	(6, 19)
Clinical integration	BAC is rarely incorporated into routine cardiovascular risk assessment or management pathways.	Not applicable; clinical outcome benefits from BAC-guided management have not been established.	Mammography may provide an opportunity for cardiovascular risk identification without additional imaging or radiation exposure.	Absence of an established referral pathway or evidence-based management algorithm	(5, 6, 19)
Patient perspective	Most surveyed women preferred to be informed when BAC was detected.	95.8% wanted BAC information included in their care, and 76.6% preferred direct communication from the radiology department.	Reporting may facilitate patient–clinician discussions regarding cardiovascular risk assessment.	Preferred communication pathways may vary among health systems and populations.	(69)
Incremental risk stratification	BAC provided additional risk information beyond conventional cardiovascular risk factors in a prospective cohort.	Hard ASCVD: adjusted HR, 1.51 (95% CI, 1.08–2.11); global CVD: adjusted HR, 1.23 (95% CI, 1.002–1.52); overall NRI, 0.12 (95% CI, 0.03–0.14); clinical NRI, 0.11 (95% CI, 0.01–0.22)	BAC may function as a women-specific risk-enhancing imaging marker.	Incremental value requires confirmation in independent and clinically diverse cohorts.	(7, 70)
Potential clinical impact	BAC assessment may identify women who warrant formal evaluation of conventional cardiovascular risk factors.	Not applicable; no prospective intervention trial has demonstrated that BAC-based screening or treatment reduces cardiovascular events.	BAC detection could prompt blood pressure, lipid, diabetes, and global cardiovascular risk assessment.	The appropriate clinical response to BAC severity remains undefined.	(7)

“Not applicable” indicates that the corresponding evidence concerns clinical practice or implementation status rather than a directly estimable diagnostic or prognostic metric.

ASCVD, atherosclerotic cardiovascular disease; BAC, breast arterial calcification; CI, confidence interval; CVD, cardiovascular disease; HR, hazard ratio; NRI, net reclassification improvement.

Currently, BAC reporting remains inconsistent and non-routine. Radiologists typically describe BAC as “present” or “absent” without standardized quantification or explicit cardiovascular risk implications (6, 19). This inconsistency stems from absent professional guidelines, historical perception of BAC as benign, and subjective manual quantification methods. Consequently, BAC information rarely translates into actionable cardiovascular interventions.

Patient surveys demonstrate strong preference for BAC communication, with 95.8% wanting notification and 76.6% preferring direct radiology department contact (74). This highlights the importance of transparent communication and BAC's potential to empower proactive cardiovascular health management. However, acceptance by radiologists and physicians requires further investigation and education.

Incorporating BAC into cardiovascular risk models may improve risk stratification, particularly in hypertensive women. Traditional tools such as the Framingham Risk Score and Pooled Cohort Equations (75) may underestimate cardiovascular risk in women, whereas BAC may improve model calibration and ASCVD risk reclassification (7). Because BAC is available from routine mammography, its assessment could support more intensive lifestyle modification, earlier pharmacotherapy, or closer clinical monitoring in selected high-risk women. However, prospective studies are required to determine whether BAC-guided risk assessment and intervention reduce cardiovascular events.

### 6.2 Existing knowledge gaps and future research directions

Despite compelling evidence supporting the association between BAC, hypertension, and CAD, several critical knowledge gaps necessitate further research to unlock BAC's clinical potential.

Current literature lacks large-scale prospective studies establishing causal relationships between BAC and future cardiovascular events. While cross-sectional and retrospective studies demonstrate strong associations, they cannot prove causality or temporal sequences. Future prospective cohort studies with long-term follow-up are essential to determine whether BAC presence and progression independently predict incident CAD, MACE, or cardiovascular mortality beyond traditional risk factors. Although hypertension, diabetes, and other cardiovascular risk factors should be managed according to established prevention guidelines, there is currently no direct evidence that improved blood pressure, glycemic, or lipid control reduces or reverses BAC (5, 6). The effect of statin therapy on BAC also remains unknown. Studies of coronary atherosclerosis suggest that statins may promote calcification of coronary plaques while reducing or stabilizing overall atheroma burden (76); however, these findings cannot be directly extrapolated to BAC, which predominantly represents medial arterial calcification. Longitudinal studies using serial mammographic BAC measurements are therefore needed to determine whether BAC progression is modified by cardiovascular risk-factor control or specific pharmacological therapies.

BAC quantification standardization remains a critical challenge. Various methods currently exist, from simple presence/absence reporting to semi-quantitative scales and continuous calcium mass measurements (19). This lack of unified, validated scoring systems hinders study comparability and clinical application. Future research should develop and validate standardized, reproducible, clinically meaningful quantitative BAC scoring systems for widespread adoption, crucial for integrating BAC into clinical guidelines and conducting robust multi-center trials.

Although AI shows promise for automated BAC detection and quantification, several challenges remain. Models require rigorous multicenter validation across different mammography systems and patient populations (21–23), because algorithms trained on single-institution datasets may not generalize to other clinical settings. Safety, robustness, algorithmic bias, data privacy, transparency, and interpretability must also be addressed (70–72). Explainable artificial intelligence (XAI), which provides insight into model outputs, may improve clinician trust and facilitate clinical implementation (70). Further research should clarify the relationships of BAC with blood pressure variability, micro-inflammation, microvascular dysfunction, and myocardial perfusion. The mechanisms through which blood pressure fluctuations and systemic inflammation influence BAC formation remain uncertain. Advanced imaging studies may help define the cardiovascular consequences of different degrees of BAC and identify potential therapeutic targets.

Research into intervention strategies specifically targeting medial calcification is crucial. Current prevention focuses on traditional risk factors, but direct calcification therapies are limited. Understanding molecular pathways in hypertension-induced medial calcification, such as GLS1-mediated glutamate pathways (29) or Klotho-FGF-23 axis (32), could enable targeted pharmacological interventions. Exploring existing medications' impact on BAC development represents an important clinical trial area (37).

Future research must establish causality, standardize quantification, validate AI tools rigorously, and explore complex pathophysiological interactions driving BAC, translating it from an imaging finding into a powerful biomarker for personalized cardiovascular risk management.

## 7 Conclusion

BAC is an increasingly recognized women-specific imaging marker associated with hypertension, CAD, and adverse cardiovascular outcomes. Automated detection and quantification using AI may improve the reproducibility and clinical integration of BAC assessment during routine mammography. However, prospective validation, standardized quantification, and evaluation of its incremental clinical value are still required before BAC can be routinely incorporated into cardiovascular risk assessment and management.
